# COVID-19 Vaccines: How Efficient and Equitable Was the Initial Vaccination Process?

**DOI:** 10.3390/vaccines11010011

**Published:** 2022-12-20

**Authors:** Jai K. Das, Hsien Yao Chee, Sohail Lakhani, Maryam Hameed Khan, Muhammad Islam, Sajid Muhammad, Zulfiqar A. Bhutta

**Affiliations:** 1 Institute of Global Health and Development, Aga Khan University, Karachi 74800, Pakistan; 2 Center of Excellence in Women and Child Health, Aga Khan University, Karachi 74000, Pakistan; 3 Global Health Research Center and Division of Natural and Applied Sciences, Duke Kunshan University, Kunshan 215316, China; 4 Center of Global Child Health, The Hospital for Sick Children, Toronto, ON M5G 1X8, Canada

**Keywords:** COVID-19, vaccines, vaccine rollout, vaccine equity, COVAX, AVAT

## Abstract

With nearly 11 billion doses of the COVID-19 vaccine being administered, stark differences in the vaccination rates persist. Vaccine distribution initiatives such as COVAX and African Vaccine Acquisition Trust (AVAT) were formed to ensure equitable vaccine delivery. This review evaluates the initial COVID-19 vaccination efforts and the impact of different vaccine distribution initiatives on equitable vaccination coverage in the early phase. We conducted a descriptive and trend analysis with sub-groups by various context parameters of data on COVID-19 vaccination from December 2020 till February 2022, from four public databases including UNICEF, WHO, COVID-19 Task Force and Our World in Data to examine COVID-19 vaccine distribution progress and the contributions of vaccine procurement initiatives. We found that High Income Countries (HICs) had much higher vaccination rate (78.4%) than Lower-Middle-Income Countries (LMICs) (55.5%) and Low-Income Countries (LICs) (10.9%). Large differentials (>80% to <10%) in the vaccination rates of eligible population of adults in LMICs and LICs existed. Differentials in the total vaccine doses delivered to each country ranged from 355.6% to 4.8% of the total population. In LICs, 53.3% of the total doses were obtained via COVAX, 30.9% by bilateral/multilateral agreements, 6.5% by donations and 3.8% by AVAT. In LMICs, 56.4% of total vaccines procured were via bilateral/multilateral agreements, 21.4% by COVAX, 4.2% by donations and 0.5% by AVAT. COVAX delivered 1 billion doses by January 2022 which constituted 53.2% and 21.4% of procured doses in LICs and LMICs. In LICs and LMICs, 6.5% and 4.2% of total doses were acquired through donations while 30.9% and 56.4% of doses were purchased. Despite global efforts, significant disparities were present in COVID-19 vaccination efforts amongst countries of different income groups. Future efforts should focus on addressing vaccine inequities explicitly and in improving global vaccine distribution.

## 1. Introduction

The severe acute respiratory syndrome coronavirus 2 (SARS-CoV-2) is responsible for the outbreak of the COVID-19 disease and was first detected in Wuhan, China in late December 2019 [1]. Beginning in early 2020, the quest to find COVID-19 vaccines proceeded with cautious optimism. However, by December 2020, through unprecedented scientific innovation and collaboration, vaccines Astra Zeneca and Pfizer-BioNTech became among the first COVID-19 vaccine to be approved for emergency use [2]. The expedited development of COVID-19 vaccines can be attributed to enormous global scientific collaboration and innovation, spurred by public financing leading to novel vaccine manufacturing processes.

The need for equitable access to COVID-19 vaccines was identified very early and led to the creation of COVID-19 Vaccines Global Access (COVAX) initiative in April 2020. The COVAX initiative is jointly directed by the Global Vaccine Alliance (Gavi), the Coalition for Epidemic Preparedness Innovations (CEPI) and the World Health Organization (WHO). The COVAX initiative was formed with the intention of coordinating international resources to aid in the development and manufacturing of the different COVID-19 vaccines while providing Lower-Middle-Income Countries (LMICs) and Low-Income Countries (LICs) with equitable access to COVID-19 tests, therapies, and vaccines by serving as a buyers and distributor club either through self-financing options or through donations [3]. The distribution plan of vaccines through COVAX can be broken down into three stages. Stage 1 included providing enough vaccine doses for all member countries to vaccinate 3% of their population where healthcare and social workers would be prioritized. Stage 2, designed to occur in parallel with stage 1, allowed for countries to purchase or receive enough vaccines to vaccinate 20% of their population. The final stage (stage 3) would only begin after all COVAX participating countries have vaccinated at least 20% of their population and would aim to vaccinate the remaining 50% of people for a target of 70% population coverage. In addition to COVAX, other vaccine distribution channels include the African Vaccine Acquisition Trust (AVAT), which was created in July 2021 with the aim of complementing COVAX’s procurement and distribution objectives to attain a target immunization of 60% of Africa’s population [4].

Toward the end of 2021, reports estimated that greater than half the global population had received at least the first dose of the COVID-19 vaccine [5,6]. However, despite the apparent speed of vaccine development and delivery, the death toll due to the disease continued to rise [7], raising concerns as to the adequate distribution and delivery of vaccines. In early 2022, the vaccine hoarding phenomenon was apparent; lower-income countries were suffering as higher-income countries were reserving and purchasing second and third doses [8,9,10]. Emergence of the Omicron variant in developed regions led to an increment in the urgent procurement of booster doses and worsening of the distribution gap [6].

Global access to COVID-19 vaccines remains an ongoing source of global health insecurity and limitation of equal access to healthcare. Recent studies have evaluated this from various aspects of vaccine development, production, allocation, deployment, and costs to achieve equitable access [11,12,13], but the thorough global analysis of inequities and impact of COVAX in the initial phase of global vaccine distribution efforts has been lacking. This review aims to examine the early global vaccination efforts and the impact of COVAX and other vaccine distribution initiatives on global access and equitable coverage.

## 2. Methods

### 2.1. Study Design

We conducted a quantitative analysis of secondary data to explore the trends, progress and equity of COVID-19 vaccine distribution and coverage globally with a special focus on LMIC and LIC. We evaluated the contribution of various procurement mechanisms including COVAX and its global roll out using publicly available information and databases during the early phase of vaccine roll-out till February 2022.

### 2.2. Data Sources

A search for COVID-19 epidemiological databases was conducted on 15 February 2022. The UNICEF Vaccine Market Dashboard [14], the WHO [15], the COVID 19 Task Force [16] and the Our World in Data (OWID) [17] repository were the key data sources for the quantitative analysis.

Epidemiological, geographical, and demographic data were extracted from the OWID and COVID 19 Task Force database. This included geographic location (country, continent, UNICEF region), demographics such as development status, population and the overall COVID-19 burden (total cases, total deaths, partial, full and booster dose vaccination coverage). The vaccination data acquired from OWID were based on official reports collected by the OWID team while data on confirmed cases and deaths were acquired through the COVID-19 Data Repository by the Center for Systems Science and Engineering (CSSE) at Johns Hopkins University [18].

For data on variables covering vaccine status, vaccine procurement details, manufacturing, and pricing, we extracted information from the UNICEF Vaccine Market Dashboard database, OWID and WHO, simultaneously incorporating data from the World Bank [4] for categorization of countries based on income status.

### 2.3. Data Analysis

For the secondary quantitative descriptive analysis, we stratified the countries into high-, upper middle-, lower middle-, and low- income countries based on the World Bank (WB) income group categorization.

Descriptive analysis of the data form WHO and the OWID database was performed on STATA Version 16.0 [19]. Data obtained covered the country name, WB categories, total vaccinations per 100 people and regional classifications. Data on vaccination proportions were compared across the nine UNICEF regions. This included a time trend analysis of vaccination coverage across all UNICEF regions depicting fully, partially and booster dose vaccinated percentage, the number of LMICs and LICs with over 50% vaccination coverage in each UNICEF region and the variation in the vaccination initiation months in LMIC and LIC worldwide.

Descriptive analysis of the UNICEF data was conducted in Microsoft Excel. The list of 213 countries and territories obtained from the database was categorized by World Bank income groups. We calculated proportions of total vaccines procured with respect to each mechanism, for the five procurement mechanism categories: COVAX, bilateral/multilateral agreements, country donations, AVAT and unknown mechanisms. Mean values of the total proportion for each procurement mechanism category were calculated and compared across the four income groups. Our outcomes of interest were the vaccine procurement status of each WB income category. This included total vaccine doses delivered as a percentage of the population per country, the number of doses delivered and contribution by the five procurement mechanisms, country level vaccine procurement mechanisms in LMICs and LICs according to UNICEF regions and the top 10 vaccine donor countries.

Trend analyses describing the specific role of COVAX in procurement initiatives were also performed. This included the time trend of vaccine doses delivered by COVAX from 1 December 2020 to 31 January 2022 and the vaccine doses delivered by COVAX as a proportion of the country’s total population.

To analyse the manufacturing power, we described countries with manufacturing capacity in each of the nine UNICEF regions, and the unit price of vaccines in USD at which a country, territory or group with manufacturing capacity agreed to supply vaccines.

## 3. Results

The analysis depicts the global, regional, and country level burden of COVID-19, coverage of vaccination, and the various procurement strategies adopted with a special focus on deliveries by COVAX agreements. In addition, it also highlights the price range at which the vaccines were bought and the countries that donated vaccines to other countries in need for the data till February 2022.

### 3.1. Vaccine Supply and Procurement

**Figure 1:**
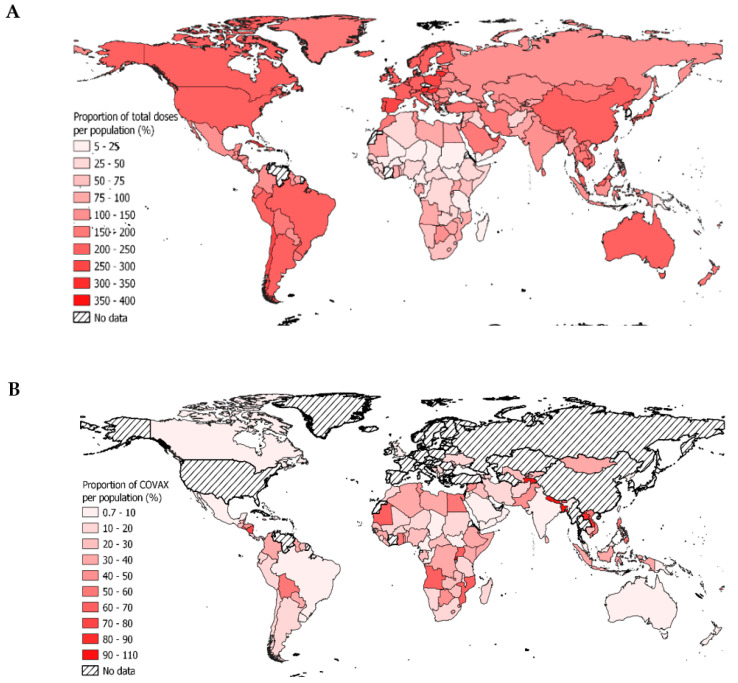
(**A**) Total vaccine doses delivered as a percentage of the population per country. (**B**) Vaccine doses delivered by COVAX as a proportion of the country’s total population.

One of the major reasons of the variation in the vaccine coverage can be attributed to the distribution of vaccine delivery. Globally, 67 countries received less than 100% and 35 countries received less than 50% of the vaccine doses as a proportion of their total population. Out of these 35 countries, 31 were LMIC and LIC most of which belonged to Western and Central Africa (n = 16) and Eastern and Southern Africa (n = 10). Figure 1A illustrates the global distribution of COVID vaccines to each country as a percentage of the country’s total population by 15 February 2022. The map shows a significant variation in the ratios ranging from 4.8% in Haiti, a LMIC to 355.6% in Gibraltar, a HIC. Following Gibraltar are Lithuania, Hungary, and Austria, HIC of Western Europe with 335.5%, 314.1% and 308.2% respectively. These countries have a relatively small population compared to the total number of vaccines they received. The least deliveries relative to total population are seen in Africa, which specifically comprises of LMIC and LIC. Congo (DRC) reports 7.55%, Cameroon reports 8.61%, and South Sudan reports 8.69% of total vaccines delivered with respect to the total population.

### 3.2. Vaccination

**Figure 2:**
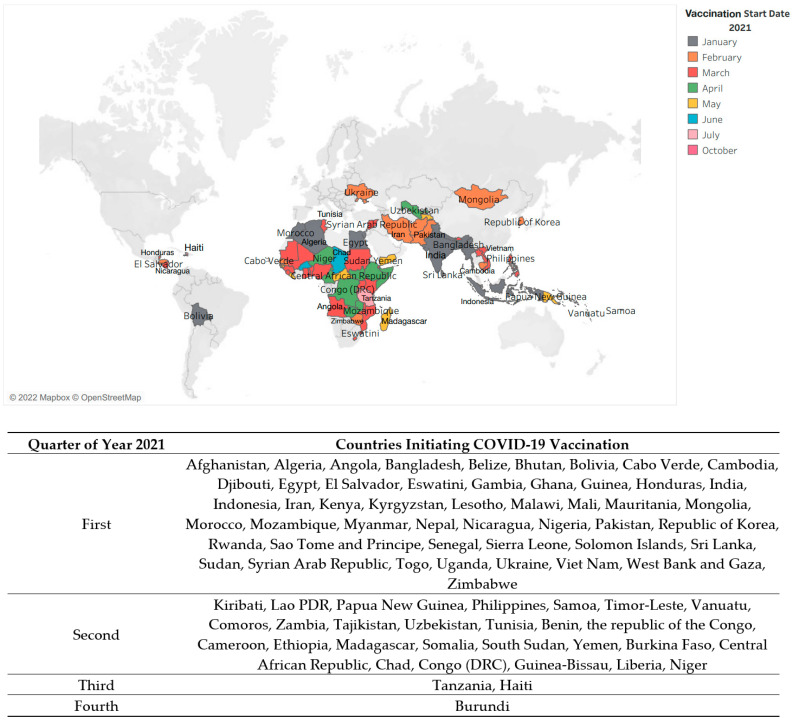
Period of Initiation of Vaccinations in LMIC and LIC.

Vaccination process began on 8 December 2020, and UK became the first country globally to initiate the campaign with its indigenous Astra Zeneca vaccine (AZ). In LMICs, India was the first country to begin the process of vaccination on 16 January 2021. Figure 2 displays the pattern of vaccination start months in LMICs after the first COVID-19 vaccine was licensed for emergency use by WHO on 31 December 2020.

As depicted in the table, the process of vaccination in LMIC began in January 2021. During the first quarter of the year; 53 LMICs and LICs managed to initiate the process in their own capacities, another 23 countries began vaccination in the second quarter of the year whereas the remaining 2 countries formally began in the third quarter of the year. Burundi was the last country to commence vaccination, in October 2021.

**Figure 3:**
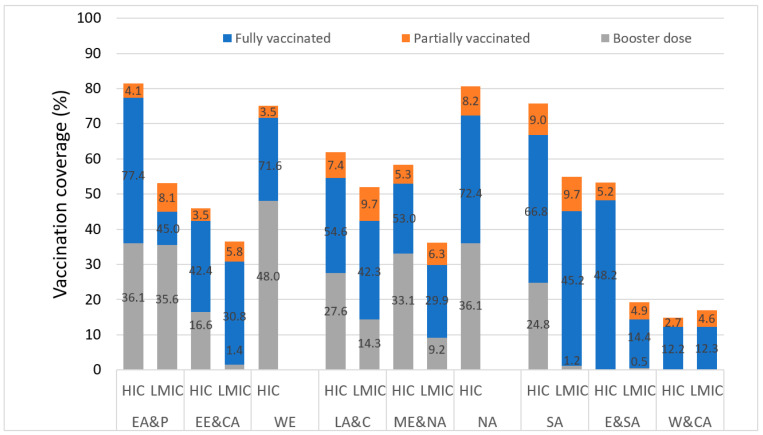
Vaccination coverage by UNICEF regions and income group as of 15 February, 2022. HIC: High Income Country; LMIC: Low- and Middle- Income Country; EA&P: East Asia and Pacific; EE&CA: Eastern Europe and Central Asia; WE: Western Europe; LA&C: Latin America and Caribbean; ME&NA: Middle East and North Africa; NA: North America; SA: South Asia; E&SA: Eastern and South Africa; W&CA: Western and Central Africa.

By 15 February 2022, approximately 62% of people globally had received at least one dose of vaccine. A great disparity was observed between income groups as nearly 78.4% in HIC and only 10.9% in LIC and 55.5% in LMIC were vaccinated. Figure 3 displays the current vaccination coverage with respect to full, partial and booster doses in the various UNICEF regions. It is worth noting from the figure above that the highest percentages of vaccination coverage are recorded in HIC of approximately all UNICEF regions with the highest disparity of 32.7 PMP observed in the Eastern Mediterranean Region. Across all nine regions, booster dose coverage was also lower in LMIC as compared to HIC. Booster vaccinations were lowest in the LMIC of E&SA (0.5%), SA (1.2%) and EE&CA (1.4%) regions. To date, there has been no booster vaccination in the W&CA region in both HIC and LMIC.

An in-depth picture of vaccination coverage amongst LMIC at a country level showed that Cambodia had the highest coverage, with over 80% of the population fully vaccinated against COVID-19. Vietnam and Bhutan were also reported to have more than 70% of the total population completely vaccinated. Burundi and Democratic Republic of the Congo had a near absent vaccination coverage, while 23 other countries shown had less than 10% of the population fully vaccinated. Only 14 out of 75 LMIC and LIC reported a fully vaccinated proportion of greater than 50%. Table S1 shows the distribution of fully vaccinated countries in each UNICEF region.

### 3.3. Vaccine Procurement and Financing

**Figure 4:**
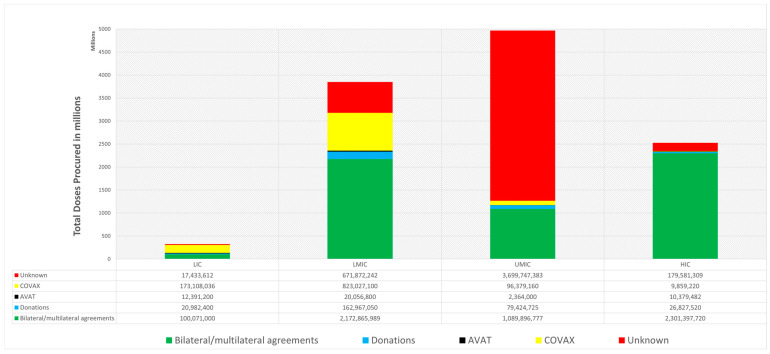
Doses delivered by the five procurement mechanisms stratified by World Bank Income Regions.

Around the globe, vaccination procurement occurred via several procurement mechanisms, including bilateral/multilateral agreements, donations, COVAX, AVAT and unknown mechanisms. Worldwide, approximately 11.7 billion doses were procured by 15 February 2022. Figure 4 demonstrates the doses procured via each of the five mechanisms across all the World Bank income groups. As evident from the figure, UMIC secured the greatest number of doses (4,967,812,045) followed by LMIC (3,850,892,181), HIC (2,528,045,251) and LIC (323,987,522).

In the LIC, 53.3% of doses were procured by COVAX, 30.9% by bilateral/multilateral agreements, 6.5% by donations, 5.4% by unknown mechanisms and 3.8% by AVAT. Simultaneously, in the LMIC, 56.4% of total vaccines procured were via bilateral/multilateral agreements, 21.4% by COVAX, 17.5% by unknown mechanism, 4.2% by donations and 0.5% by AVAT. At the same time, in UMIC, 74.5% of total vaccines were procured by unknown mechanism, 21.9% was procured by bilateral/multilateral agreements, 1.9% by COVAX and less than 0.1% by AVAT. Concurrently, in HIC, 91.0% were procured by bilateral/multilateral agreements, 7.1% by unknown mechanisms, 1.1% by donations and 0.4% each by AVAT and COVAX.

A deeper insight into the LMIC according to the UNICEF Regions (Figure S3) showed that countries in the South Asian region such as India procured the highest number of doses (1,691,626,143). The major proportion of vaccines in India was procured by bilateral/multilateral agreement totaling to 1,165,176,072. Countries in the East Asia and Pacific region such as Kiribati, Samoa and Vanuatu secured the least number of doses and a significant proportion of doses were procured by COVAX in these countries.

### 3.4. COVAX

Vaccine deliveries through COVAX began in January 2021. A total of 23 HICs, 42 UMICs, 51 LMICs and 25 LICs benefited from COVAX. Globally, there was a very gradual increase in COVAX deliveries during the first half of 2021, from a total of 171,185,389 doses in January 2021 to 255,165,664 doses in June 2021. The steepest rise in deliveries was seen between November and December 2021. COVAX has delivered a total of approximately 1.1 billion vaccine doses worldwide at the time of writing. This was short of COVAX’s initial goal of distributing 2 billion doses by the end of 2021 where the billionth dose supplied by COVAX was only distributed on 15 January 2022 [20]. LMICs received the maximum deliveries through COVAX, with a cumulative total of 804,142,460 doses delivered as of January 2022 (Figure S1).

The map (Figure 1B) depicts the doses delivered by COVAX globally, as a proportion of each country’s population. On a regional level analysis of LMIC, the highest proportion of COVAX deliveries to total population was observed in Latin America and Caribbean region (0.44) whereas the lowest was observed in South Asian region (0.16). On a country level analysis, the highest proportion of COVAX deliveries to total population was seen in Samoa (LMIC), Rwanda (LIC), Saint Lucia (UMIC), and Maldives (UMIC) accounting to approximately 170 each. The lowest proportion was seen in India (0.72), an LMIC with a population of approximately 1.4 billion where COVAX delivered only 10 million vaccine doses.

### 3.5. Donations

Data suggests that approximately 2.5% of all the vaccines procured globally were by donations from various countries. Out of the total donations, LMIC received 55.25% and UMIC received 27.29% of doses. Table S2 shows top 10 countries that donated vaccines globally.

### 3.6. Price Range of Vaccines

Amongst the various mechanisms of procurements, there were direct buying agreements between countries, territories, and groups. Figure S2 shows a range of prices per dose from USD 4 to USD 40 for which manufacturers agreed to supply vaccines to 41 countries, territories and groups.

## 4. Discussion

Development of the COVID-19 vaccine was a huge scientific leap forward in terms of its use of mRNA technology, improved manufacturing processes and its speed of regulatory approval. This resulted in it becoming a key strategy in achieving the herd immunity needed to end the pandemic. However, stark differences existed and continue to persist in the vaccination rates between countries of different income groups and geographic regions.

As evident from the results of our study, approximately 78.4% of the population in HIC and only 10.9% and 55.5% population in LIC and LMIC respectively were vaccinated by 15 February 2022. Out of the 75 LMIC listed, only 14 countries had reported a fully vaccinated proportion of greater than 50%. This variation in the statistics can be owed to the discrepancy in deliveries with respect to the population of the country. In all, 31 LMIC and LIC received fewer than 50% doses as a proportion of their total population, most of which were in the African regions. On the other hand, HICs such as Gibraltar, Lithuania and Hungary had access to more than 300% doses with respect to their population. It is worth noting that, to date, HIC and UMIC have procured a total of 7.47 billion vaccines followed by LMIC 3.85 billion, and LIC only 320 million. Despite having the capacity to manufacture and purchase vaccines, HIC and UMIC procured a sizeable number of doses through COVAX, summing to approximately 105 million doses. At the same time, only approximately 170 million doses were delivered to LIC by COVAX. As a whole, COVAX’s contribution to global vaccination efforts were far short of its 2 billion dose target where the facility only delivered 910 million doses in 2021.

This lower than anticipated delivery highlights one of several shortcomings in the COVAX mechanism. Firstly, the facility was over reliant on one source—the Serum Institute of India (SII) as a primary supplier of vaccines which abruptly stopped exporting COVID-19 vaccines in March 2021. COVAX’s impact was also blunted by supply-chain issues, vaccine nationalism, hoarding of vaccines by HICs, vaccine hesitancy and lack of urgency from local governments in administering the vaccines [21]. In addition, many vaccine doses obtained by COVAX through donations were close to expiring. This resulted in some LICs rejecting COVID-19 vaccine doses [22] where COVAX has had to preferentially allocate doses to countries with sufficient vaccine deployment infrastructure and delivery systems and thus undermine the basic principle of equitable distribution [23]. In recent times, COVAX has also become increasingly reliant on donations from other countries to meet vaccine delivery targets. Such strategies, while effective in the short-term, do not provide a reliable supply of vaccines as donations can be suddenly suspended in the case of acute outbreaks or emergence of new variants in donor countries. Recent evidence concludes that in future, without considerable reforms to the global system of vaccine governance, COVAX alone will not be sufficient to tackle the persisting global inequity of vaccine access [24,25].

In addition, further limitations also exist in the financial model of COVAX. From the onset, COVAX did not synergize all global vaccine procurement channels where its members were allowed to strike bilateral deals with vaccine manufacturers while simultaneously being part of the COVAX initiative. Such practices resulted in a fragmented system of vaccine procurement where COVAX engaged in direct competition with HICs to acquire vaccines on the open market. Such competition allowed rich countries easier and faster access to vaccines where pharmaceutical companies expedited deliveries to HICs who paid a premium. The result of such competition is reflected in Figure 4 where bilateral/ multilateral agreements accounted for the procurement of 91% of the total vaccine doses acquired by HICs. This limitation is exacerbated by the payment structure of COVAX in how the contributions of member countries was not proportional to national wealth. Member countries were instead grouped into two distinct buyer’s clubs called the “COVAX Facility” and the “Gavi Advance Market Commitment for COVID-19 Vaccines (GAVI AMC). The COVAX Facility consisted of HICs and HMICs while the 92 poorest members of COVAX were grouped into the GAVI AMC. With all members only being required to contribute to their individual buyer’s club, this effectively removed any responsibility for HICs and HMICs to provide financial assistance to GAVI AMC thus resulting in a lack of funding. The COVAX has also been suggested to aid financialization of global health and privileging the corporate aspects [26].

COVAX’s initial target of supplying 20% of vaccines to a country was ineffective in mitigating the spread of the virus, and a higher initial target of 40%-50% had been estimated by previous studies to be the minimum vaccination rate for controlling the virus [27]. WHO expanded their strategy to ensure that all countries had vaccinated 70% of their populations by mid-2022, with an interim 40% by end 2021 [28]. In a recent evaluation of the first-year impact of vaccinations, an estimated 19.8 million deaths from COVID-19 were potentially averted as a result of vaccinations. However, these reductions were specifically noticed in high-income regions. In low-income countries, particularly those that did not reach the 40% target, the impact was minimal. Had the targets been reached in these countries, the vaccine impact on disease mitigation is estimated to have been almost doubled [5]. Impouma et al. [29] reported that by the end of June 2022, the WHO African region had received 625 million doses of COVID-19 vaccines, of which 66% were from COVAX. These doses however, represented only 40% of the doses needed to fully vaccinate 70% of people in all countries, thus compounding the trajectory of regional inequity highlighted by our findings in January 2022.

In terms of improvements to COVAX’s implementation, the issue of vaccine hoarding by HICs could have been prevented if COVAX had increased their early investment into the research and development of COVID-19 vaccines. This would have granted them more leverage when trying to purchase vaccines at later stages of the pandemic and ensured a continued supply of vaccines to LMICs and LICs. COVAX could also have emulated the policies adopted by the European Union’s COVID-19 vaccine buyer’s club by setting in place agreements for all member countries to purchase vaccines through the initiative instead of directly from the manufacturers. That aside, the various domestic issues faced by LMICs and LICs, could have largely been avoided if COVAX had better diversified their resource allocation. These include supporting improvements to the health and supply chain infrastructure in these countries and actively funding community education programs that increase awareness on the importance of vaccines while actively combating misinformation to reduce vaccine hesitancy.

Currently, with greater capacity for vaccine development globally, COVAX should continue investing in the development of a range of novel COVID-19 vaccines which would increase supply and better safeguard against future variants of the virus. COVAX should also place additional emphasis on supporting research efforts for vaccines that are less sensitive to variations in temperature, have a longer storage life and which are easier to administer, such as oral vaccines. Additionally, COVAX should also continue to advocate for vaccine developers to share vaccine intellectual property through the WHO’s Coronavirus Treatment Acceleration Program (CTAP) mechanism. This can be conducted in tandem with allocating resources to support the establishment of domestic vaccine manufacturing capabilities in various countries around the world through technology transfer initiatives. Such initiatives involve original vaccine manufacturers partnering with local or regional pharmaceutical companies to produce vaccines domestically to safeguard supply and increase the speed of vaccination rollout with the agreement between AstraZeneca and Siam Bioscience in Thailand being a prime example [30]. That said, the initiative should actively seek partnerships and investments with local governments in improving cold-chain infrastructure to increase governmental buy-in and ensure the smooth and prompt rollout of vaccines when acquired.

On a global scale however, the COVID-19 pandemic has raised awareness to the importance of international cooperation in responding to issues of human health and highlighted the need for a comprehensive international agreement that outlines the various steps and procedures in responding to health issues of global concern. Such an agreement would allow for greater levels of international collaboration while also having the added effect of increasing accountability for governments around the world. An international agreement of this nature would thus ensure that sufficient resources and systems are devoted to limiting the effects of future pandemics.

## 5. Conclusions

Both vaccine charity and vaccine diplomacy have failed to ensure the equitable delivery of vaccines. Despite facilitation efforts by COVAX and other mechanisms, considerable differentials in the distribution of COVID-19 vaccines were observed around the world. Future initiatives on the allocation of pandemic vaccines worldwide should be based on complete and precise knowledge of population dynamics, the area specific burden of disease, capacity to purchase and deliver vaccines. These initiatives should also focus on innovative strategies for vaccine financing, research and vaccine development. The world in such global crisis situations should form partnerships that offer common platforms for policies, implementation and roll out of vaccination plans. While seemingly utopian, these measures are a cornerstone for global health security as the human and the economic implications of inequitable pandemic response and vaccination distribution will ultimately be faced by all nations.

## Data Availability

All data used in the study is open-sourced and are publicly available on the sources listed below. No new datasets were generated during this study. UNICEF Vaccine Market Dashboard: https://www.unicef.org/supply/covid-19-vaccine-market-dashboard (accessed on 15 February 2022); COVID 19 Taskforce: https://data.covid19taskforce.com/data (accessed on 15 February 2022); WHO: https://app.powerbi.com/view?r=eyJrIjoiMWNjNzZkNjctZTNiNy00YmMzLTkxZjQtNmJiZDM2MTYxNzEwIiwidCI6ImY2MTBjMGI3LWJkMjQtNGIzOS04MTBiLTNkYzI4MGFmYjU5MCIsImMiOjh9 (accessed on 15 February 2022); Our World in Data (OWID): https://ourworldindata.org/coronavirus (accessed on 15 February 2022).

## Supplementary Materials

The following supporting information can be downloaded at: https://www.mdpi.com/article/10.3390/vaccines11010011/s1, Figure S1 Time trend analysis of vaccines delivered by COVAX, Figure S2 Unit price of vaccines for which a manufacturer agreed to supply vaccine in USD, Figure S3 Country level vaccine procurement mechanisms in LMIC and LIC according to UNICEF regions, Table S1 LMIC and LIC in UNICEF regions with vaccination coverage of 50%, Table S2 Top 10 vaccine donor countries around the globe.
