# Supplementary material for: COVID-19 Vaccines: How Efficient and Equitable Was the Initial Vaccination Process?
Source: Vaccines (Basel). 2022 Dec 20;11(1):11. doi: 10.3390/vaccines11010011 (PMC9862832; doi:10.3390/vaccines11010011)
Supplement: Supplementary file 1 [file vaccines-11-00011-s001.zip › Figure S3 Country level vaccine procurement mechanisms in LMIC and LIC according to UNICEF regions.pdf]

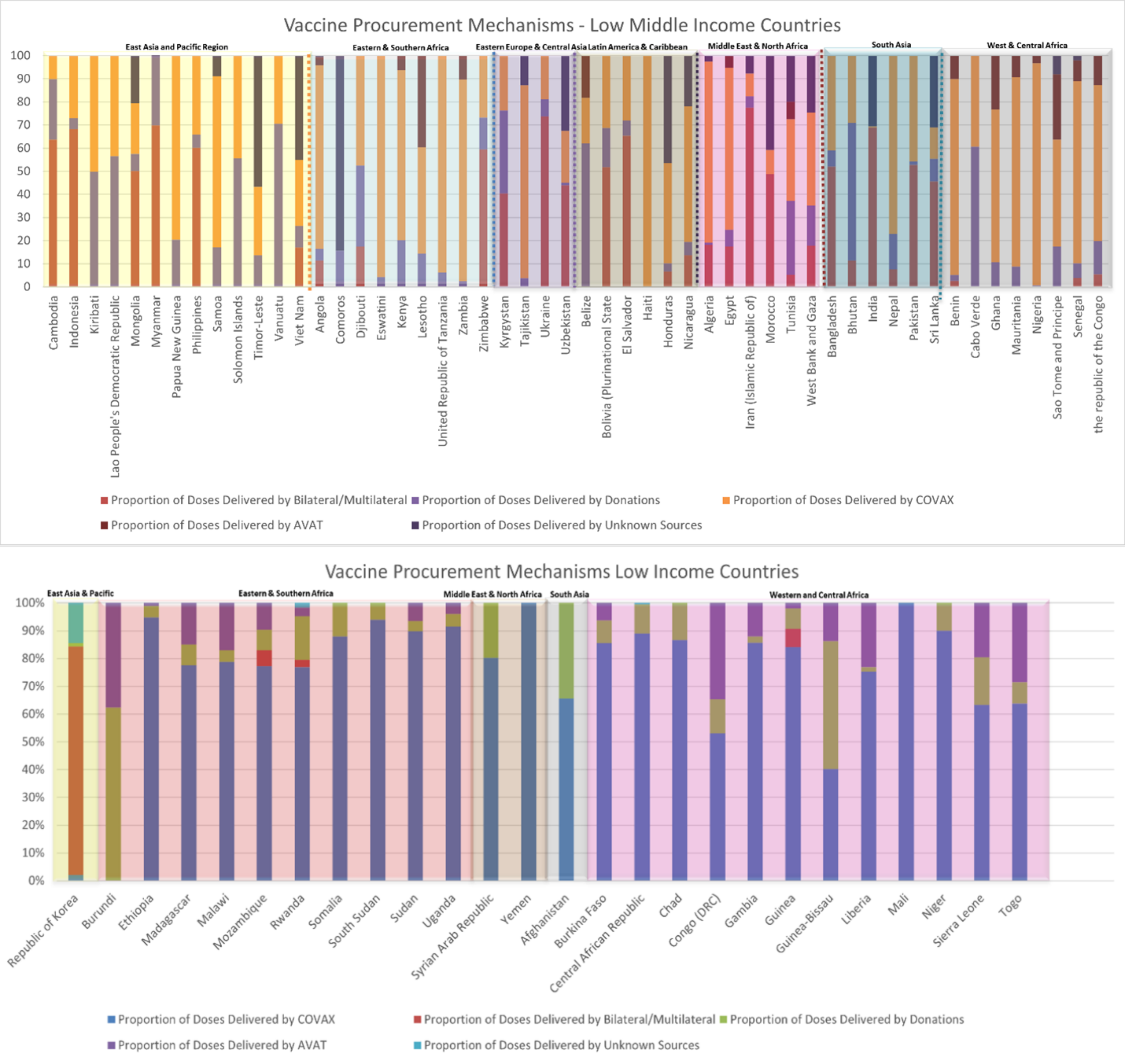

Figure S3: Country level vaccine procurement mechanisms in LMIC and LIC according to UNICEF regions
