# Supplementary material for: COVID-19 Vaccines: How Efficient and Equitable Was the Initial Vaccination Process?
Source: Vaccines (Basel). 2022 Dec 20;11(1):11. doi: 10.3390/vaccines11010011 (PMC9862832; doi:10.3390/vaccines11010011)
Supplement: Supplementary file 1 [file vaccines-11-00011-s001.zip › Table S1 LMIC and LIC in UNICEF Regions with Vaccination Coverage of 50_.pdf]

Table S1: LMIC and LIC in UNICEF regions with vaccination coverage of >50%

UNICEF Regions	Fully vaccinated ">50%"	Out of total
East Asia and Pacific	5	12
Eastern and Southern Africa	1	19
Eastern Europe and Central Asia	0	5
Latin America and Caribbean	2	5
Middle East and North Africa	2	6
South Asia	3	6
West and Central Africa	1	22
**Total**	**14**	**75**
