# Supplementary material for: COVID-19 Vaccines: How Efficient and Equitable Was the Initial Vaccination Process?
Source: Vaccines (Basel). 2022 Dec 20;11(1):11. doi: 10.3390/vaccines11010011 (PMC9862832; doi:10.3390/vaccines11010011)
Supplement: Supplementary file 1 [file vaccines-11-00011-s001.zip › Table S2 Top 10 vaccine donor countries around the globe.pdf]

Table S2: Top 10 vaccine donor countries around the globe

Donor	Donated doses
United States of America	185,505,850
China	127,475,340
Germany	111,604,163
France	74,216,230
Japan	64,183,490
United Kingdom of Great Britain and Northern Ireland	57,363,860
Italy	50,339,570
Spain	49,878,550
Canada	26,785,800
Netherlands	23,824,415
