# Supplementary figures and images for: COVID-19 Vaccines: How Efficient and Equitable Was the Initial Vaccination Process?
Source: Vaccines (Basel). 2022 Dec 20;11(1):11. doi: 10.3390/vaccines11010011 (PMC9862832; doi:10.3390/vaccines11010011)

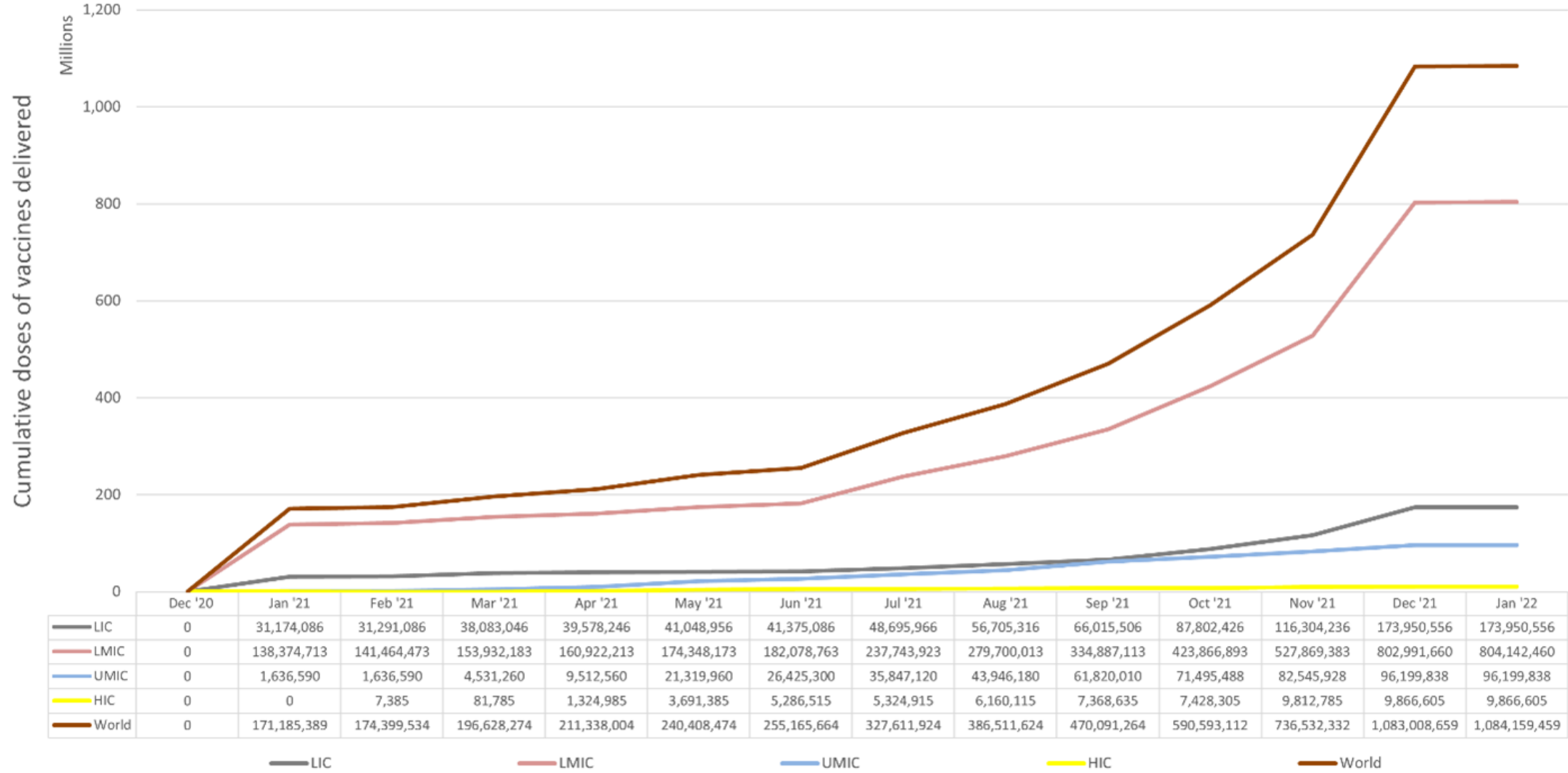

**Figure S1: Time trend analysis of vaccines delivered by COVAX**

Supplement: Supplementary file 1 [file vaccines-11-00011-s001.zip › Figure S1 Time trend analysis of vaccines delivered by COVAX.pdf]

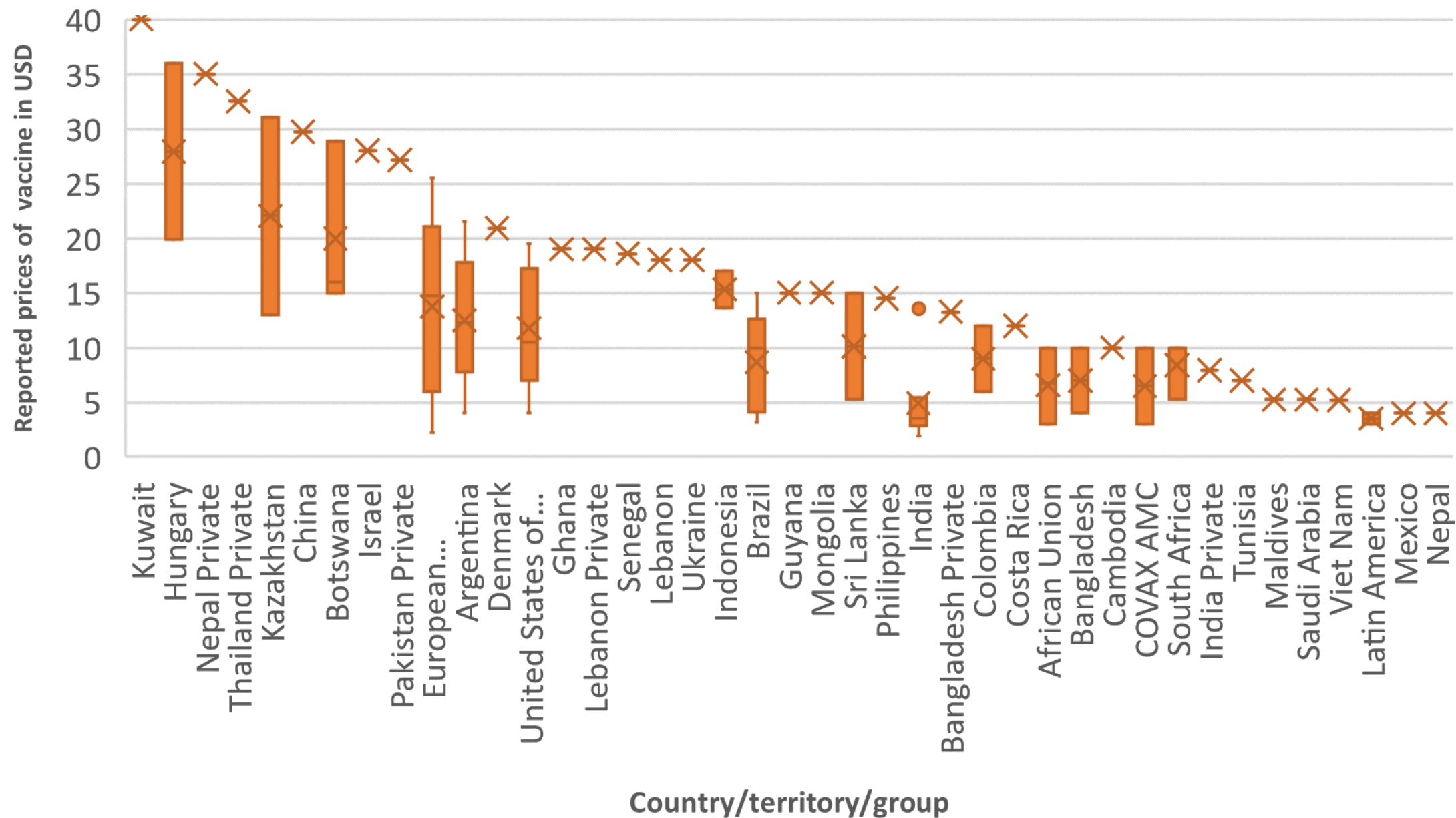

Figure S2: Unit price of vaccines for which a manufacturer agreed to supply vaccine in USD

Supplement: Supplementary file 1 [file vaccines-11-00011-s001.zip › Figure S2 Unit price of vaccines for which a manufacturer agreed to supply vaccine in USD.pdf]
